# Notes and News

**Published:** 1895-03-30

**Authors:** 


### Notes and News.

Collected and Communicated.

HOSPITAL MEETINGS.

National Hospital for Paralysed and Epileptic.—Biennial Festival Dinner, Tuesday, April 22nd, Whitehall Rooms, Hotel Metropole.

A new wing of the National Lying-in Hospital, Holles Street, Dublin, was opened last week by the Archbishop of Dublin.

Earl Compton, M.P., will preside at the annual dinner of the Royal Hospital for Incurables, West Hill, Putney Heath, on Thursday, May 23rd, at the Albion Tavern, Aldersgate Street.

At the opening of the Medical Congress on Tuberculosis, which met at Coimbra this week, a telegram was despatched to Dr. Koch to congratulate him upon his work of investigation into the causes of tubercular disease.

A sale of work is to be held at Westminster Hospital on Wednesday and Thursday, April 3rd and 4th, in aid of the general funds. Lord and Lady Knutsford have consented to open the sale at three o'clock on Wednesday. Most of the articles to be sold have been made by the nurses of the hospital, and as they are very reasonably marked, lovers of bargains should not fail to make their way to Westminster on one or both of the days named. Nurses will find many articles adapted for their special needs.

The death of Corney Grain, whose name has been for so long a household world amongst us, has been a real and personal sorrow to hundreds. It is only natural that this feeling should find expression in a desire to perpetuate his memory in some lasting fashion, and a suggestion made by a correspondent in another column, might well and fittingly be accepted by those to whom the idea commends itself. It is that a "Corney Grain" cot be endowed in the Great Ormond Street Children's Hospital in memory of one whose genial fun has given pleasure to many thousands of children. Surely no better monument could be devised than this, and we hope that the proposal may meet with prompt and ready response.

Mr. Thomas Smith, a late vice-president of the Royal College of Surgeons, and senior-surgeon to St. Bartholomew's Hospital, has been appointed Surgeon-Extraordinary to the Queen, in the room of the late Sir William S. Savory, Bart.

The Dental Hospital of London has been promised a further donation of £1,000 from Mr. Henry J. Harben, J.P., one of the vice-presidents, in addition to £500 already contributed by him towards the building fund of the new hospital in Leicester Square upon the condition that £13,000 is contributed by the public within a certain time.

The gale of Saturday and Sunday last has been responsible for much damage to life and property up and down the country. At the Walsall Hospital much injury was caused by the fall of two chimney stacks upon the roof. One ward entirely collapsed, and its occupants were buried beneath the débris. No lives were lost, but several of the patients were badly hurt.

On Saturday last the annual meeting of the Hospital Saturday Fund was held at the Mansion House. Mr. Reginald D. Acland presided, and Canon Scott Holland, Canon Fleming, the Rev. H. R. Wakefield, Dr. Theodore Acland, and many others were present. It was stated that £17,609 had been distributed during the past year amongst the medical charities of London. The street collections had decreased, whereas there was a considerable increase in the workshop collections. A resolution was proposed and adopted in approval of the principle of weekly subscriptions from working men. The street collection for 1895 has been arranged to take place on Saturday, July 13th.

The Ipswich Corporation has, according to a contemporary, concluded an arrangement with a local yacht club to lend for use as a club house a floating hospital procured by the town a short time since, at some expense, for cases of cholera, or other infectious disease. By this most extraordinary and undesirable arrangement no rent is to be paid by the club, but the vessel is to be maintained in good order, and given up at an hour's notice. The plea of economy put forward by the Corporation can in no way justify such a proceeding, and it is to be hoped that the people of Ipswich, for whose benefit and safeguarding the hospital exists, will take steps to see that it is restored, and kept sacred to its proper uses, and ready at all times of emergency.

We congratulate Dr. Holman, the able treasurer of Epsom College, upon the fact that from July 1st, 1895, the resident pensioners will be granted an increase in their pensions (*i.e.*, to £30) in lieu of residence, and all the buildings of the College will then be devoted to the use of the scholars. This reform has been urgently called for for many years, and it has been made possible by the provisions of the will of the late Dr. Bowen, of Melbourne. To make the step absolutely secure in the future, it will be necessary for the Governors of the College to give a continuous and generous support to its benevolent branch, which will no doubt benefit largely by the dinner which will be held on May 15th at the Queen's Hall, Langham Place, the Right Hon. A. J. Balfour, M.P., in the chair. Any offers of contributions or co-operation will be esteemed by the treasurer, Dr. Holman, at the office, 37, Soho Square, W.

